# Evaluation of mature soybean pods as a food source for two pod-sucking bugs, *Riptortus pedestris* (Hemiptera: Alydidae) and *Halyomorpha halys* (Hemiptera: Pentatomidae)

**DOI:** 10.1371/journal.pone.0176187

**Published:** 2017-04-21

**Authors:** M. Mahbubur Rahman, Un Taek Lim

**Affiliations:** 1 Bangladesh Agricultural Research Institute, Rahmatpur, Barisal, Bangladesh; 2 Department of Bioresource Sciences, Andong National University, Andong, Republic of Korea; 3 Institute of Agricultural Science and Technology, Andong National University, Andong, Republic of Korea; College of Agricultural Sciences, UNITED STATES

## Abstract

*Riptortus pedestris* (Fabricius) and *Halyomorpha halys* (Stål) cause injury to soybeans by piercing and sucking pods and seeds. Growers believe that new damage decreases near to harvest despite the occurrence of these bugs at that time. As this question has never been assessed, we evaluated two diets: a) mature soybean pods (dried shell + dried soybean seeds) and b) dried soybean seeds for the two bugs by assessing their biological, behavioral, and morphological attributes on each diet in laboratory. While nymphs of both species were able to develop and adults able to reproduce on the tested diets, bugs fed on pods had longer development times and 2.2 to 5.0 times higher mortality rates than bugs fed on seeds. Furthermore, adult longevity of *R*. *pedestris* and *H*. *halys* fed on pods was 8.4 and 7.5 days shorter, respectively, than that of bugs fed on seeds. However, pod feeding had no effect on adult fecundity or egg viability. In a behavioral choice test, adult *R*. *pedestris* preferred seeds over pods and probed seeds longer than pods. On average, adult *H*. *halys* also preferred seeds over pods, although 15.6% of *H*. *halys* showed the reverse, preferring pods over seeds. The proboscis length and estimated depth of stylet penetration into the host tissue of both nymphs and adults of both species was much greater than the thickness of the pod shell, suggesting that mouthpart structure does not explain the negative effects of pods vs. seeds. In conclusion, mature soybean pods were found to be a suitable food source for both *R*. *pedestris* and *H*. *halys* despite some negative effects, and thus careful attention should be paid to the population levels of these two bugs approaching harvest to reduce economic damage in soybean.

## Introduction

Soybean, *Glycine max* (L.) Merr., is one of the most important legumes. Soybean yields are reduced by several hemipteran insect pests that feed on the pods and seeds [1]. Among hemipterans, alydids and pentatomids are considered the most important soybean pests, causing nutritional and yield losses [2–5]. Hemipteran pests of soybean feed through punctures made in the pod by inserting piercing-sucking mouthparts and forming a stylet sheath to convey downward saliva containing digestive enzymes for tissue breakdown and upward for the extraction of plant liquids [6, 7]. The other function of the stylet sheath is to deliver substances that influence wound healing, defense signaling pathways, and the emission of volatile substances by the plant [8, 9]. On seeds, feeding punctures appear as minute dark spots [10], and feeding on seeds reduces yield, quality, and germination [11–13]. Transmission of microorganisms and common yeast spot disease by *Riptortus pedestris* (Fabricius) and *Halyomorpha halys* (Stål) has also been reported [14, 15].

Hemipterans preferentially feed on young, tender growth and developing seeds (growth stages R4-R6 in soybean) [16, 17], but peak infestation of stink bugs in soybean fields generally occurs at the mid to late pod filling stage (stages R5-R7) [16, 18, 19, 20, 21]. However, Kim and Lim [22] and our preliminary observations found *R*. *pedestris* and *H*. *halys* present in soybean fields at the mature pod stage (R8) in fall, suggesting that this stage of soybean is also suitable for feeding by these bugs. Bae et al. [23] reported that exposure of mature soybean pods to *R*. *pedestris* led to lower seed quality and yield and even reduced germination. Of hemipteran bugs found in soybean fields in Korea, *R*. *pedestris* and *H*. *halys* are the most common [3]. *Halyomorpha halys* feeds more and causes more severe damage to soybean than *R*. *pedestris* [23] even though *R*. *pedestris* is generally regarded as the most common soybean pest in Korea. Bae et al. [23] suggested that *H*. *halys* may change *R*. *pedestris’* pest status on leguminous crops under certain environmental conditions (temperature and availability of spring food). *Halyomorpha halys* has already become an important pest of several host plants including leguminous crops and fruit trees in Korea, and has invaded the United States [24, 25].

Many studies have assessed soybean damage by stink bug during the soybean cultivation period [3, 11, 12]. However, the comparative damage to soybean pods and seeds late cultivation period has never been assessed. It is a common practice to keep soybean stands in the field for a longer period of time at the matured seed stage (R8) for better drying. Hence, understanding the effect of bug feeding on mature soybean pods contributes to management decisions just prior to harvest. Growers believe that bug populations cause less damage during once hard pod shells have developed.

In this study, we compared mature soybean pods containing seeds and dried soybean seeds removed from pods as food sources for the two bugs by assessing biological, behavioral, and morphological attributes in the laboratory.

## Material and methods

### Ethics statement

This study did not involve any endangered or protected species. Insect collection was carried out at the experimental station of Andong National University. All necessary permits were obtained to collect insects from experimental fields.

### Rearing of test species

*Riptortus pedestris* adults and nymphs were collected from soybean fields at the experimental station of Andong National University, Andong, Republic of Korea, and reared in the laboratory according to the method described by Kim et al. [26]. Nymphs and adults were held separately in acrylic cages (40L×40W×40H cm) with one door and three meshed screens in the lateral sides for ventilation at 21.9–31.1°C, 35.0–68.8% RH, and a 16L:8D h photoperiod. Water dissolved ascorbic acid (2.0 g/L) in cotton and dried soybean seeds were provided as food sources to adult and nymph *R*. *pedestris*. Red kidney bean plants (*Phaseolus vulgaris* L.) with cotyledonous leaves were given additionally to nymphs. Four pieces of gauze were placed in both upper and bottom corners of each cage as oviposition substrates. Wild *R*. *pedestris* males were frequently added to the adult rearing cages to avoid inbreeding depression.

*Halyomorpha halys* adults and nymphs were collected from mustard, sesame, and soybean fields at the experimental station of Andong National University in 2014 and reared in the laboratory according to the method described by Kim et al. [27]. Adults and nymphs were brought into the laboratory and placed inside acrylic cages described above. Ascorbic acid (2.0%), dried soybean and peanut (*Arachis hypogaea* L.) seeds, and plants of red kidney bean and pepe (*Peperomia obtusifolia* A. Dietr.) were provided as food sources. Insects were reared at 21.9–31.1°C, 35.0–68.8% RH, and a 16L:8D h photoperiod. Field collected *H*. *halys* males were frequently added to adult cages to avoid inbreeding depression.

### Diet evaluation

(Exp. 1a) Effect of test diets on *R*. *pedestris* nymphal development. To obtain newly emerged nymphs to begin rearing, eggs (< 24 h old) were collected from the adult rearing cages and kept in insect breeding dishes (10D×4H cm, 310102, SPL, Pocheon, Korea; Petridish type with an opening lid having a meshed screens in the upper sides for ventilation) until they hatched. After hatching, each first instar nymph was reared in an insect breeding dish and provided with one of the two treatments (mature pods or seeds). Either three soybean pods containing 2–3 seeds/pod or 6–8 dried soybean seeds were provided, together with cotton soaked with ascorbic acid (2.0%) in each treatment. *Riptortus pedestris* experimental units consisted of bugs reared on either mature intact soybean pods (dried shell + dried soybean seeds) or dried soybean seeds as treatments. Even though we didn’t measure the dryness, dried pods collected from experimental soybean fields in 2014 were used for all experiments. During rearing, nymphs were held at 25.6 ± 0.1°C, 47.2 ± 0.1% RH, and a 16L:8D h photoperiod in a growth chamber (DS-11BPL, Dasol Scientific Co., Ltd, Hwaseong-si, Gyeonggi-do, Korea). Nymphal instar (as reflected in size and their exuvia) and survival were observed daily. We did not test mature pod shell (after removal of seed) in life table studies because, with this diet, none of 12 *R*. *pedestris* adults reproduced in a preliminary study.

(Exp. 2a) Effect of test diets on *R*. *pedestris* adults that were reared as nymphs on each test diet. When *R*. *pedestris* nymphs reared on the test diets became adults, each resultant female was paired with a male from the same rearing food in another insect breeding dish. As an oviposition substrate a small piece of gauze was provided on the edge of the upper part of the breeding dish, and the breeding dishes were held under the same environmental conditions as described above. Pre-ovipositional period, life time fecundity, hatch rate of eggs produced, and longevity of adult females were recorded. Seventy first-instar nymphs were tested for each food source.

(Exp. 3a) Effect of test diets on *R*. *pedestris* adults that were reared as nymph on soybean seeds. Fifth-instar nymphs of *R*. *pedestris* were collected from the rearing cage and kept in an insect breeding dish with soybean seeds and ascorbic acid as food sources. When nymphs became adults, individual females were paired with males and held in same diet treatments described above (bugs were fed on pods from the beginning of adult stage) and their reproduction and egg hatch rate observed at 25.6 ± 0.1°C and 47.2 ± 0.1% RH. As *R*. *pedestris* nymph usually become very low in density during late cultivation period (R8), mature soybean pods would be exploited by *R*. *pedestris* adult only. Thus this experiment was designed to assess effect of mature soybean pods on adult reproduction, i.e., rate of egg laying, pre-ovipositional period, fecundity, and egg hatch rate.

(Exp. 1b) Effect of test diet on *H*. *halys* nymphal development. For all experiments with *H*. *halys*, the treatments (food types) were the same as for *R*. *pedestris* (mature pods and seeds removed from pods). Five egg masses (containing approximately 30 eggs each) of *H*. *halys* that had been oviposited within the previous 24 h were randomly chosen from the rearing colony. Then those masses were placed individually in breeding dishes, and held at 25.0 ± 0.1°C and 52.2 ± 0.1% RH in a growth chamber. When eggs hatched, 10 first instars were randomly chosen from each of the five egg masses and placed as a group in one insect breeding dish (the same as described above) at the same temperature and RH and fed with either mature soybean pods or separated seeds, supplemented in both cases with ascorbic acid (2.0%). When first instar nymphs molted into the second instar, these were placed individually into breeding dishes, 50 with each on the two test foods. Nymphs were checked daily for development and mortality. Cotton soaked with ascorbic acid (2.0%) was changed every day, and food sources and the breeding dish were changed twice a week. Molts were noted by the presence of exuvia and nymphal instar characteristics as outlined by Hoebeck and Carter [25].

(Exp. 2b) Effect of test diet on *H*. *halys* adults that were reared as nymphs on each test diet. When nymphs reared on the test diets became adults, females were paired with males from the same diet in new breeding dishes. A small piece of gauze was provided on the edge of the upper part of the breeding dish as an oviposition substrate, and the breeding dishes were held under the same environmental conditions as described above. For each female, we recorded the preovipositional period, life time fecundity, hatch rate and longevity. Fifty individuals of the first instar nymphs were tested for each food sources. To obtain more adults for reproduction experiments, we reared 30 more nymphs on each of the two test foods under the same conditions as in experiment (1b) but without checking their development and mortality.

(Exp. 3b) Effect of test diets on *H*. *halys* adults that were reared as nymphs on soybean seeds. Fifth instar nymphs were collected from the rearing cage and two individuals per breeding dish were kept with soybean seeds and ascorbic acid. When nymphs became adults each female was paired with a male and held at 25.9 ± 0.1°C and 48.7 ± 0.1% RH in a growth chamber in another breeding dish with one of the two food sources and their lifetime fecundity and hatch rate was recorded.

### Life history parameters

To construct a fertility life table for both species, the following parameters were recorded and calculated [28]:

1. Number of females per group (Treatments) (*NF*_*g*_). The number of mated females in each group (*g = 1*, *2*, *3*,*………G*)
2. Initiation of adult stage (*t0*_*gi*_). Time span between the day when that female become an adult and the day of oviposition that originated a female *i* (*i = 1*, *2*, *3*,........*n*_*g*_) of a group g.
3. Longevity (*tf*_*gi*_). Time span between the day of oviposition of adult female *i* of a group *g* is born and the day of its death.
4. Number of eggs (*NEGG*_*gix*_). The number of eggs laid by female *i* of group *g*, in each pivotal age *x* (the female age plus 0.5).
5. Total eggs (*NEGG*_*gx*_). Total number of eggs laid by all females of group *g*, at the pivotal age *x* was calculated as
  $${NEGG}_{gx}=\sum_{i=1}^{n_{g}}{NEGG}_{gix}$$
6. Ratio of females (*SR*_*g*_). As sex determination of eggs and nymphs is not possible, the proportion of female eggs was calculated by taking a sample of *Ng* eggs from each group and observing them until adult emergence, at which point the number of female adults (*NAF*_*g*_) was divided by the total number of adults (*NA*_*g*_). The ratio of females in each group, called the sex ratio, was expressed as
  $${SR}_{g}=\frac{{NAF}_{g}}{{NA}_{g}}$$
7. Immature stage survivorship (*SURV*_*g*_). Percent of offspring females alive until adult emergence, called preimaginal survivorship, was calculated by dividing the number of females alive until adult emergence by *N*_*g*_ (the number eggs taken from each group to determine the sex ratio). It was calculated as
  $${SURV}_{g}=\frac{{NA}_{g}}{N_{g}}$$
  The information listed above is used to estimate parameters related to the population growth potential in each group. The size of population at time *N(t)* was calculated as [28]
  $$N(t)=N_{0}\times e^{r_{m}\times t}$$
  where *N(t)* is the size of population at time *t*; *N*_*0*_ is the initial population size, and *r*_*m*_ is intrinsic rate of increase.
8. Net reproduction rate (*R*_*0*_). The net contribution from each female to the next generation, which is expressed as the total of female offspring per female during the entire oviposition period [28]. The number of female offspring coming from all females of the group *g* at each pivotal age *x* (*m*_*gx*_) is necessary to calculate *R*_*0*_ as follows
  $$m_{gx}={NEGG}_{gx}\times {SR}_{g}$$
  This is also needed to calculate cumulative female survivorship (*l*_*gx*_) as
  $$l_{gx}={SURV}_{g}\times \frac{{NSF}_{gx}}{{NF}_{g}}$$
  The net contribution at pivotal age *x* in group *g* (*R*_*0 gx*_) calculated as
  $${Ro}_{gx}=l_{gx}\times m_{gx}$$
  Therefore, the net reproduction rate of each group is calculated as
  $${Ro}_{g}=\sum_{{x=xo}_{g}}^{\Omega_{g}}{Ro}_{gx}$$
9. Mean generation time (*T*). Time span between the birth of individuals of a generation and that of the next generation is called mean generation time (*T*) [28], and is calculated as
  $$T_{g}=\frac{\sum_{{x=xo}_{g}}^{\Omega_{g}}x\times l_{gx}\times m_{gx}}{\sum_{{x=xo}_{g}}^{\Omega_{g}}l_{gx}\times m_{gx}}$$
10. Intrinsic rate of increase (*r*_*m*_). The intrinsic rate of increase is the rate of population growth [28], derived from an exponential growth model considering *t* = *T*_*g*_ and *R*_*0g*_ = *Nt*_*g*_*/N*_*0*_ [28] and calculated as
  $$r_{mg}=\frac{Ln({Ro}_{g})}{T_{g}}$$
11. Doubling time (*Dt*). Doubling time is the time span required for doubling of the initial population [28], calculated as
  $${Dt}_{g}=\frac{Ln(2)}{r_{mg}}$$
12. Finite rate of increase (*λ*). Finite rate of increase is the multiplication factor of the original population at each time period [28]. The rate of increase for each group *g* was calculated by as
  $$\lambda_{g}=e^{r_{mg}}$$

### Choice responses of both *R*. *pedestris* and *H*. *halys* between the test diets

To prepare insects for a choice experiment between the two diets, fifth instar nymphs of each species were collected from the rearing colonies and then held in breeding dishes and checked daily. After emergence (< 24 h), adult females were starved individually in breeding dishes provided with ascorbic acid only for 12–14 h before use in experiments.

For *R*. *pedestris*, treatments were two mature soybean pods containing seeds (T1) and four extracted soybean seeds (T2), both of which were placed in a single insect breeding dish (same as used above) with 5 cm between the two foods. Newly emerged adult females were placed in each breeding dish, and its food exploitation behaviors observed. The behaviors was measured as i) the choice rate (initial selection), ii) the time taken to encounter the food source (latency to response), iii) the frequency of giving-up first choice without feeding, iv) the handling time before probing, v) probing frequency, vi) probing duration, and vii) the interval between probings. These observations were made for 1 h at 26.6 ± 0.01°C and 40.8 ± 0.3% RH in the laboratory.

For *H*. *halys*, the same choice test was run, as described above for *R*. *pedestris*, except that observations were made for a longer period of 3 h due to their longer probing duration. The observations were made at 26.6 ± 0.01°C and 39.2 ± 0.1% RH in the laboratory.

### Morphological observations of rostrum length vs. pod thickness

To determine if pod thickness was a barrier for bug feeding, we compared pod thickness to rostrum lengths for all instars (except 1st instar) and adults of both species. First instar nymph was excluded as it is non-feeding stage for both *R*. *pedestris* or *H*. *halys*. To obtain insects for these observations, eggs of *R*. *pedestris* or *H*. *halys* were collected from the laboratory colony as described above and kept in an insect breeding dish. The progression of development from first instar to adult was monitored daily to obtain specimens of desired stages for measurements. Ten individuals of instar 2 through 5, plus adult males and adult females for each species were examined. Within 24 h of reaching a specific instar, within 24 h randomly selected bugs were collected and placed in a falcon tube (50 ml) containing ethyl alcohol (94.5%). When all bugs were dead, individual specimens were examined under stereomicroscope and an ocular micrometer was used to measure the rostrum length, according to the method of Esquivel [29].

**Fig 1:**
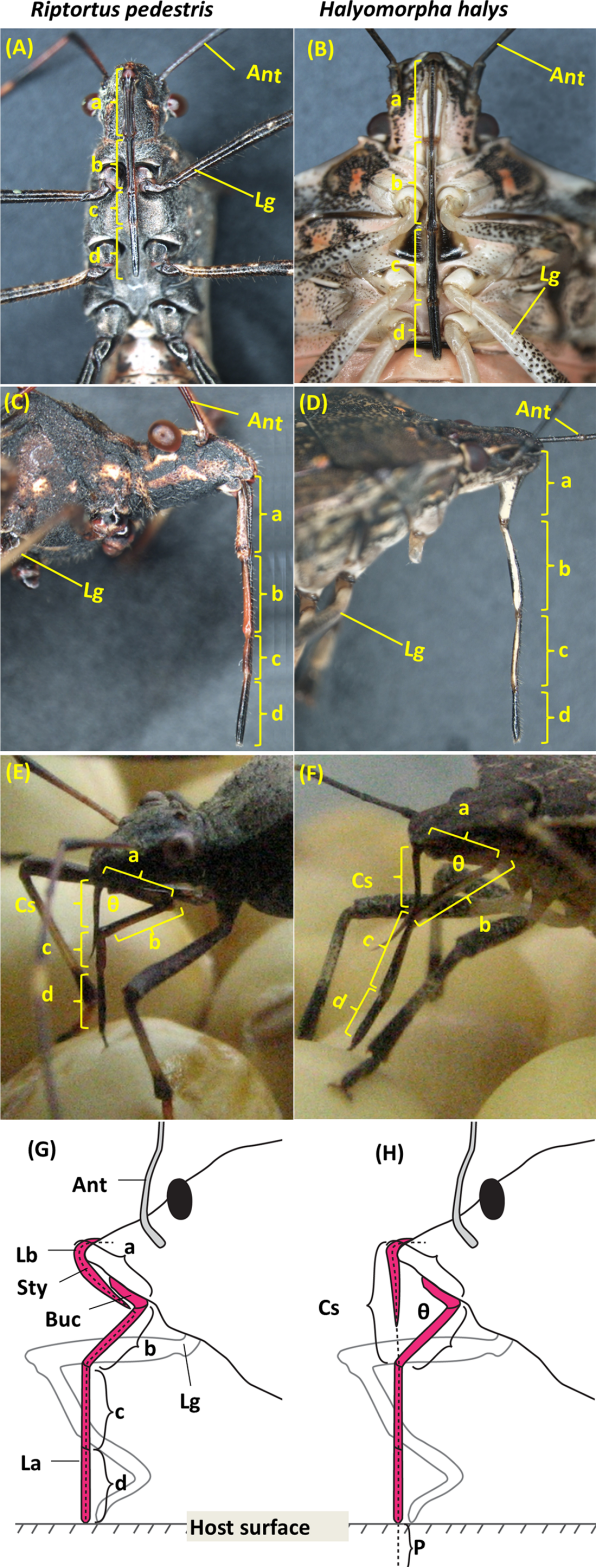
**Rostrum of *Riptortus pedestris* and *Halyomorpha halys***; ventral view (A, B), lateral view (C, D) during feeding (E, F), and schematic diagram of stink bug head with rostrum in probing (G) and feeding postures (H). rostrum a-d, length of segments 1–4, respectively; Ant, antenna; Buc, bucculae; Cs, calculated span; La, labium; Lb, labrum; Lg, Leg; P, penetration depth into host tissue; Sty, stylet bundle; ⱷ, angle at segment 2 and bucculae.

The rostrum of hemipterans is 4-segmented, where segments 1 and 4 are located proximal and distal to the head, respectively (Fig 1). When the insects are at rest, the rostrum lies parallel on the ventral surface of their body. The proximal end of segment 2 can articulate at the junction with the bucculae. During probing and feeding, the distal end of segment 2 swings away from the insect body, thereby forming an angle between bucculae and the proximal end of segment 2 (Fig 1). The proximal end of segment 3 articulates with the distal end of segment 2, aligning itself with segment 4. Thus, the stylet bundle remains lying within segments 2 to 4 when insects assess good food sources. To reach or achieve feeding posture, the proximal end of segment 2 articulates at the juncture with the bucculae (or segment 1; Fig 1) enabling the insect to maneuver segment 2 to various angles. These maneuvers create unknown spans between the head apex and proximal end of segment 3, and these spans directly affect penetration depth. The span distance is derived by the angle created at the bucculae and the proximal end of segment 2. To calculate the created unknown span from the apex of the head to the proximal end of segment 3 (Fig 1), rostral segments 1 and 2 were measured for incorporation into Eq 1 of Esquivel [29] as:

$$\mathrm{Calculated}\;\mathrm{span}\;\left(\mathrm{cs}\right)={\left(a^{2}+b^{2}\text{-}2\mathrm{ab}\;\cos\theta \right)}^{1/2}$$ (1)

where a = length of segment 1 as previously described, b = length of segment 2, θ = anterior angle at junction of distal end of the bucculae with proximal end of segment 2, and cs = calculated span (Fig 1).

Values (i.e., cs) obtained from Eq 1 were incorporated into Eq 2 of the model to estimate stylet penetration depth (P). The stylet penetration depth (P) for insects with a given angle at the juncture of segment 2 and the bucculae (Fig 1) is calculated as:

Styletpenetrationdepth(P)=∑(a+b)-cs (2)

where a = length of segment 1, b = length of segment 2, cs = calculated span from Eq 1, and P = stylet penetration depth (Fig 1).

We also measured the rostrum segments 3 and 4 to calculate total rostrum length. Length of individual segment of the rostrum was critical for implementing the model. We measured each segment length for nymphal stages of both species, as well as those of male and female adults.

For comparison to rostrum lengths, we measured the thickness of the shell of mature soybean pods that were collected from fields in 2014, and of the seeds that were removed from the pods. Ten pod shells (n = 10) were cut into small pieces, and their thickness was measured under stereomicroscope using an ocular micrometer.

### Statistical analyses

Nymphal mortality rates were compared between the diets using a Chi-square test with a post-hoc multiple comparison test analogous to Tukey’s test [30]. Development times, preoviposition periods, fecundity, and adult longevity were analyzed with *t*-tests.

We used the jackknife method proposed by Meyer et al. [31] to estimate the variance for *r*_*m*_. Algorithms for jackknife estimation of the means and variances, and the construction of confidence intervals are described only for *Ro*. Similar procedures were used for the other parameters like *r*_*m*_, *T*, *Dt*, and *λ*. All data related to fertility life tables were entered into a computer program (LIFETABLE.SAS) [28] and analyzed using SAS 9.3 [32]. Comparisons of all parameters between pod- and seed-fed bugs were done with *t*-tests. The *P* values corresponding to the two-tailed (P_*T*_), upper-tailed (P_*U*_), and lower-tailed (P_*L*_) *t*-tests were calculated using a cumulative Student *t* distribution (Probit) based on Sidák equality [33].

Choice rates in the behavioral observations were compared by Chi-square tests and a post-hoc multiple comparison test analogous to Tukey’s test use to separate means. Time taken to choose a food source, handling time before probing, probing duration, and the intervals between probing were analyzed by *t*-test.

Rostrum lengths were analyzed by single factor analysis of variance (ANOVA) and differences in mean length were determined by Tukey’s test using Proc MIXED of SAS 9.3 [32]. The model included mean rostrum length as the response variable with developmental stage of the insect as a fixed effect and replicates within stage as random effects. Similarly, differences in stylet penetration distance were determined using Tukey's test using Proc MIXED. The response variable was stylet penetration distance while stages and angles at the junction of segments 1 and 2 were fixed effects. Data were also analyzed independently to compare estimated stylet penetration by angle within stage and by stage within angle using two factors ANOVA, and mean separation of stylet penetration estimates by angle within stage and by stage within angle was done by Tukey’s test using Proc MIXED.

## Results

### Diet evaluation

Exps. 1a and 1b. Effect of test diets on nymphal development in *R*. *pedestris* and *H*. *halys*. Nymphs of both *R*. *pedestris* and *H*. *halys* fed on mature soybean pods had longer development times than nymphs fed on exposed seeds. Similarly, nymphal mortality of both species was higher when fed on mature pods than exposed seeds; mortality of *R*. *pedestris* nymphs fed on pods and seeds was 21.4 (pods) and 4.3% (seeds) and for *H*. *halys* nymphs was 44.0 (pods) and 20.0% (seeds). In both species, pod feeding affected 2nd instars most. As first-instar do not feed on plant tissue, their survivorship was not significantly affected by diet treatment (Table 1).

**Table 1:** Effect of soybean pod on the development and sex ratio of *Riptortus pedestris* nymphs (n = 70) and *Halyomorpha halys* (n = 50).

Insect	Parameters	Food source	Development time (days ± SE)						Sex ratio (ϘϘ)
			1st	2nd	3rd	4th	5th	1st-5th	
*Riptortus pedestris*	Development time	Pod	3.0 ± 0.0	3.3 ± 0.1	3.0 ± 0.1	3.8 ± 0.1	6.5 ± 0.1	19.6 ± 0.2	0.49
		Seed	3.0 ± 0.1	2.8 ± 0.0	3.0 ± 0.0	3.4 ± 0.1	6.1 ± 0.1	18.3 ± 0.1	0.51
		*t/Z* *P*	0.24 0.809	4.97 <0.001	0.82 0.412	3.44 0.001	3.78 < 0.001	7.39 < 0.001	0.01 1.000
	Mortality (%)	Pod	0.0	15.7	2.9	0.0	2.9	21.4	
		Seed	0.0	2.9	0.0	0.0	1.4	4.3	
		*Z*		2.02	0.82		0.16	2.29	
		*P*	-	0.020	0.415	-	0.877	0.006	
*Halyomorpha halys*	Development time	Pod	4.8 ± 0.2	12.1 ± 0.1	7.9 ± 0.2	8.4 ± 0.2	11.1 ± 0.1	44.4 ± 0.5	0.52
		Seed	4.7 ± 0.3	11.7 ± 0.1	7.2 ± 0.2	8.1 ± 0.1	10.4 ± 0.1	42.1 ± 0.4	0.55
		*t/Z* *P*	0.47 0.64)	2.24 0.030	2.23 0.030	0.79 0.43	2.17 0.030	2.98 0.003	0.13 0.896
	Mortality (%)	Pod	0.0	30.0	10.0	2.0	2.0	44.0.	
		Seed	0.0	12.0	6.0	2.0	0.0	20.0	
		*Z*	-	1.96	0.37	0.71	0.00	2.36	
		*P*	-	0.049	0.712	0.48	1.00	0.018	

Exps. 2a and 2b. Effect of test diets on *R*. *pedestris* and *H*. *halys* adults that were reared as nymphs on each test diet. Pod feeding at the nymphal stage on a particular diet did not adversely affect reproduction parameters (preoviposition period, fecundity and hatch rate) in either species. However, pod feeding did significantly reduce adult longevity, by 15.0% in *R*. *pedestris* and 7.5% in *H*. *halys* when they fed on pods as nymphs (Table 2).

**Table 2:** Effect of soybean pod on reproduction and longevity of *Riptortus pedestris* and *Halyomorpha halys* adults that were reared as nymphs on test diet.

Insect	Food	Percent of bugs laid egg	Preoviposition period (days ± SE)	Life time fecundity	Longevity (days ± SE)	Hatch rate
*Riptortus pedestris*	Pod	100.0 (27/27)	6.1 ± 0.2	146.4 ± 8.9	47.1 ± 2.2	0.89 (110/124)
	Seed	100.0 (27/27)	5.6 ± 0.2	156.6 ± 6.4	55.4 ± 1.0	0.90 (122/135)
	(*Z/t*, *P*)	(-)	(1.91, 0.062)	(0.99, 0.329)	(23.71, < 0.001)	(0.23, 0.816)
*Halyomorpha halys*	Pod	91.3 (21/23)	14.1 ± 0.4	146.9 ± 6.7	41.7 ± 0.8	0.82 (201/245)
	Seed	96.6 (28/29)	13.4 ± 0.4	155.3 ± 4.5	49.2 ± 1.0	0.82 193/234)
	(*Z/t*, *P*)	(0.21, 0.836)	(1.67, 0.062)	(1.64, 0.107)	(6.15, < 0.001)	(0.01, 0.995)

Exp. 3a and 3b. Effect of test diets on *R*. *pedestris* and *H*. *halys* adults that were reared as nymphs on soybean seed. Similarly, when bugs were fed on pods from the beginning of the adult stage (when nymphal food was soybean seed), none of these reproduction parameters were negatively affected in either species (Table 3).

**Table 3:** Effect of soybean pod on reproduction of *Riptortus pedestris* and *Halyomorpha halys* adults that were reared as nymphs on soybean seeds.

Insect	Food	Percent of bugs laid egg	Preoviposition period (days ± SE)	Fecundity^a^	Hatch rate
*Riptortus pedestris*	Pod	96.7 (29/30)	6.4 ± 0.2	78.8 ± 3.8	0.88 (106/120)
	Seed	100.0 (30/30)	6.6 ± 0.2	76.9 ± 4.0	0.91 (128/141)
	(*Z/t*, *P*)	(-)	(0.68, 0.500)	(0.55, 0.587)	(0.44 0.658)
*Halyomorpha halys*	Pod	90.0 (27/30)	14.0 ± 0.5	143.0 ± 11.4	0.82 (240/292)
	Seed	93.3 (28/30)	12.9 ± 0.9	157.4 ± 6.1	0.85 (253/298)
	(*Z/t*, *P*)	(-)	(1.17, 0.246)	(1.88, 0.066)	(0.78, 0.438)

^a^10 day fecundity for *R*. *pedestris* and life time fecundity for *H*. *halys*.

### Life table parameters for both species as affected by diet

Jackknife estimates of life table parameters (*R*_*0*_, *r*_*m*_, *λ*, *T*, and *Dt*) for both species fed on soybean pod or seed, together with their 95% CL (Table 4) of fertility parameters (with standard errors and *P* values corresponding to one- and two-tailed *t*-tests (Table 4) showed that for *R*. *pedestris*, *R*_*0*_ was higher for bugs fed on seeds than pods, while other life table parameters were not significantly different between the treatments. For *H*. *halys*, *R*_*0*_, *r*_*m*_, and *λ* were significantly higher, and *T* and *Dt* shorter, when fed on seeds than pods.

**Table 4:** Estimated parameters of life history data of *Riptortus pedestris* and *Halyomorpha halys* fed on soybean pod and seed; jackknife estimates (mean) for each pair group compared, respective standard errors (SE) and *p*-values for two tailed (*P*_*T*_), lowertailed (*P*_*L*_), and uppertailed (*P*_*U*_) *t*-test for pair wise group comparison.

Insect	Parameter	Pod	Seed	Two tailed	One tailed	
		Mean ± SE	Mean ± SE	*P*_*T*_	*P*_*U*_	*P*_*L*_
*Riptortus pedestris*	*R*_*o*_	5,645.71 ± 65.11	7,605.93 ± 51.73	< 0.001	0.999	< 0.001
	*r*_*m*_	0.22 ± 0.00	0.23 ± 0.00	0.182	0.909	0.091
	*λ*	1.25 ± 0.00	1.26 ± 0.00	0.182	0.909	0.091
	*T*	38.90 ± 0.08	39.34 ± 0.07	0.438	0.781	0.219
	*Dt*	3.12 ± 0.01	3.05 ± 0.01	0.188	0.094	0.906
*Halyomorpha halys*	*R*_*o*_	4,408.57 ± 27.45	7,008.57 ± 31.97	< 0.001	1.000	< 0.001
	*r*_*m*_	0.13 ± 0.00	0.14 ± 0.00	< 0.001	1.000	< 0.001
	*λ*	1.14 ± 0.00	1.15 ± 0.00	< 0.001	1.000	< 0.001
	*T*	63.35 ± 0.09	61.87 ± 0.06	< 0.001	0.003	0.997
	*Dt*	5.23 ± 0.00	4.84 ± 0.00	< 0.001	0.000	1.000

Net reproduction rate—*R*_*o*_ (female offspring/female), Intrinsic rate of increase—*r*_*m*_ (female offspring/female/day), Finite rate of increase - *λ* (female offspring/female/day), Mean generation time—*T* (day), Doubling time—*Dt* (day)

### Choice responses of both *R*. *pedestris* and *H*. *halys* between the test diets

Both *R*. *pedestris* and *H*. *halys* preferred soybean seeds to pods, as shown by the choice rate (Table 5). Both species probed longer when fed seeds. Interestingly, no *R*. *pedestris* gave up their first choice of pods, while 16.0% of *H*. *halys* gave up their first choice of pods. There was also no significant difference in the time taken to encounter a food, the probing frequency, or the interval between probing. *Riptortus pedestris* had longer handling times when they fed on pods, but *H*. *halys* showed no significant difference in handling times between pods and seeds (Table 5).

**Table 5:** Feeding behaviors of *Riptortus pedestris* (n = 54) and *Halyomorpha halys* (n = 32) on soybean pod for the duration of 1 and 3 h, respectively (choice test).

Insect	Food source	No. of bugs with no response	Choice rate	Time to encounter (min)	Giving-up rate of first choice	Handling time before probing (min)	Probing frequency	Probing duration (min)	Interval of probing (min)
*Riptortus pedestris*	Pod	20	0.35 (12/34)	8.6 ± 0.9	0.00	8.1 ± 0.6	1.1 ± 0.0	22.3 ± 0.9	8.3 ± 2.1
	Seed		0.65 (22/34)	9.4 ± 0.4	0.00	3.7 ± 0.2	1.3 ± 0.1	33.2 ± 1.0	5.4 ± 1.2
	(*Z/t*, *P*)		(2.18, 0.029)	(0.62, 0.630)	-	(6.41,< 0.001)	(0.81, 0.421)	(3.55, 0.001)	(1.11, 0.315)
*Halyomorpha halys*	Pod	5	0.33 (9/27)	6.2 ± 0.43	0.16	4.7 ± 0.2	1.0	118.8 ± 7.8	-
	Seed		0.67 (18/27)	11.9 ± 2.6	0.00	3.6 ± 0.4	1.0	141.9 ± 3.8	-
	(*Z/t*, *P*)		(2.18, 0.029)	(0.96, 0.340)	(1.55, 0.121)	(0.68, 0.500)	-	(2.34, 0.030)	-

### Rostrum length vs. pod thickness

Total rostrum length differed significantly among the life stages of both *R*. *pedestris* (*F*_5,59_ = 482.56, *P* < 0.001) and *H*. *halys* (*F*_5,59_ = 430.82, *P* < 0.001). The smallest rostrum length was observed in second instar nymphs (the youngest stage tested) and gradually increased with developmental stage. Female bugs had longer rostrums than males (Table 6).

**Table 6:** Mean lengths of individual rostral segments and total rostrum length of *Riptortus pedestris* and *Halyomorpha halys* (n = 10).

Insect	Life stages	Rostrum length (mm ± SE) of segment				
		1	2	3	4	Total
*Riptortus pedestris*	2nd	0.56 ± 0.02	0.60 ± 0.01	0.38 ± 0.03	0.62 ± 0.03	2.16 ± 0.04a
	3rd	0.80 ± 0.02	0.98 ± 0.02	0.44 ± 0.02	0.80 ± 0.02	3.02 ± 0.06b
	4th	0.90 ± 0.02	1.24 ± 0.02	0.70 ± 0.03	1.02 ± 0.04	3.86 ± 0.07c
	5th	1.38 ± 0.02	1.20 ± 0.03	0.76 ± 0.02	1.16 ± 0.02	4.5 ± 0.07d
	adult ♂	1.44 ± 0.02	1.52 ± 0.02	0.78 ± 0.02	1.30 ± 0.03	5.04 ± 0.05e
	adult ♀	1.54 ± 0.03	1.50 ± 0.02	0.92 ± 0.02	1.35 ± 0.02	5.31 ± 0.03f
*Halyomorpha halys*	2nd	0.52 ± 0.02	0.58 ± 0.04	0.62 ± 0.01	0.54 ± 0.02	2.26 ± 0.06a
	3rd	1.02 ± 0.04	1.38 ± 0.04	1.10 ± 0.02	0.96 ± 0.03	4.46 ± 0.03b
	4th	1.16 ± 0.03	1.52 ± 0.06	1.32 ± 0.05	1.06 ± 0.02	5.06 ± 0.03c
	5th	1.38 ± 0.03	1.76 ± 0.05	1.44 ± 0.02	1.20 ± 0.03	5.78 ± 0.06d
	adult ♂	1.68 ± 0.04	1.82 ± 0.10	1.44 ± 0.05	1.16 ± 0.02	6.10 ± 0.15e
	adult ♀	2.05 ± 0.05	2.10 ± 0.03	1.68 ± 0.03	1.24 ± 0.02	7.12 ± 0.08f

Means followed by a different letter are significantly different (α = 0.05).

Penetration depth of the stylet into host tissue (*R*. *pedestris F*_5,299_ = 1965.62, *P* < 0.001; *H*. *halys F*_5,299_ = 1081.20, *P* < 0.001) and the angle formed by the proximal end of segment 2 (*R*. *pedestris F*_4,299_ = 1456.87, *P* < 0.001; *H*. *halys F*_4,299_ = 597.10, *P* < 0.001) were all significantly different among the life stages for both species (Table 7). Overall, mean penetration depth significantly increased with life stage. Mean penetration depth of the stylets of second, fifth instar, adult male, and adult female *R*. *pedestris* was 0.48, 1.08, 1.26 and 1.31 mm, while the same was 0.47, 1.31, 1.51 and 1.78 mm for *H*. *halys*, respectively. Overall mean penetration depth also increased as the angle at the proximal end of segment 2 became more acute in relation to the ventral surface of the head. The range of estimated mean penetration depth was 0.65–1.29 mm for *R*. *pedestris* and 0.82–1.60 mm for *H*. *halys* at 90–50°. As insects matured, acute angles produced deeper penetration depth. Estimated penetration depth of adults was 3.0 and 4.0 times more than that of second instars for all angles of *R*. *pedestris* and *H*. *halys*, respectively. By way of comparison, the average (± SE) thickness of matured soybean pod shells was 0.18 ± 0.01 mm.

**Table 7:** Estimated mean penetration depth of stylet in both species when rostral segment 2 is at indicated angle (n = 10, thickness of pod shell = 0.18 ± 0.01mm).

Insect	Angle	Penetration depth of stylet in different life stage (mm ± SE)					
		2nd nymph	3rd nymph	4th nymph	5th nymph	male adult	female adult
*Riptortus pedestris*	90°	0.33 ± 0.01aA	0.51 ± 0.01aB	0.61 ± 0.01aC	0.75 ± 0.02aD	0.87 ± 0.01aE	0.89 ± 0.01aF
	80°	0.40 ± 0.01bA	0.62 ± 0.01bB	0.75 ± 0.01bC	0.91 ± 0.02bD	1.06 ± 0.01bE	1.08 ± 0.01bF
	70°	0.48 ± 0.01cA	0.74 ± 0.01cB	0.89 ± 0.02cC	1.09 ± 0.02cD	1.26 ± 0.01cE	1.29 ± 0.01cF
	60°	0.56 ± 0.01dA	0.87 ± 0.02dB	1.04 ± 0.02dC	1.27 ± 0.03dD	1.48 ± 0.02dE	1.51 ± 0.01dF
	50°	0.65 ± 0.01eA	1.00 ± 0.02eB	1.19 ± 0.02eC	1.47 ± 0.03eD	1.70 ± 0.02eE	1.75 ± 0.01eF
*Halyomorpha halys*	90°	0.32 ± 0.01aA	0.68 ± 0.01aB	0.76 ± 0.02aC	0.90 ± 0.01aD	1.02 ± 0.04aE	1.21 ± 0.02aF
	80°	0.39 ± 0.02bA	0.83 ± 0.02bB	0.93 ± 0.02bC	1.09 ± 0.01bD	1.25 ± 0.05bE	1.48± 0.02bF
	70°	0.46 ± 0.02cA	0.99 ± 0.02cB	1.10 ± 0.02cC	1.30 ± 0.02cD	1.49 ± 0.06cE	1.77 ± 0.02cF
	60°	0.54 ± 0.02dA	1.14 ± 0.02dB	1.29 ± 0.03dC	1.52 ± 0.02dD	1.75 ± 0.07dE	2.07 ± 0.03dF
	50°	0.62 ± 0.03eA	1.32 ± 0.03eB	1.49 ± 0.03eC	1.75 ± 0.02eD	2.01 ± 0.08eE	2.39 ± 0.03eF

Means followed by a different lowercase letter within a column or uppercase letter within a row are significantly different (α = 0.05).

## Discussion

Both *R*. *pedestris* and *H*. *halys* were able to develop and reproduce on mature soybean pods even though bugs fed on pods took longer to complete their development and suffered higher mortality than bugs fed on seeds. On mature soybean pods, the mortality of 2nd instars of *R*. *pedestris* and *H*. *halys* was 16.0 and 30.0%, but when bugs were fed on seeds, mortality dropped to 3.0 and 12.0%, respectively.

Previous studies have reported that hemipterans such as *Nezara viridula* (L), *Chinavia hilaris* (Say), *Euschistus servus* (Say), and *Piezodorus guildinii* (Westwood) preferred to feed on young pod stages with developing seeds (R5-R6) [16, 17]. Mature soybean pods (R8) have been found to have some negative effects on nymph mortality [34, 35], an effect which might be related with the hardness of the pod shell as well as its hairiness. However, Santos and Panizzi [35] found no significant difference in adult reproduction between alydid bugs fed on mature pods or dried seeds. Nevertheless, they also reported that adult bugs lived longer when fed on mature seeds than on mature pods, a finding similar to that of our current study.

Life tables are fundamental tools for understanding the critical life stages of arthropod development and their influence on overall population structure [36]. This is a first study to create a fertility life table of both *R*. *pedestris* and *H*. *halys* fed on a limited source of diet, thus it may not reflect real situation in the nature. Nevertheless, Acebes-Doria et al. (2016) [37] compared effect of different diets on nymphal development and adult fitness of *H*. *halys*. We demonstrated that both species can successfully survive and develop from egg to adult and reproduce on mature soybean pods as well as seeds. In the case of *R*. *pedestris*, the population growth rate (*R*_*0*_) was higher when bugs were fed on seeds than on pods, but other parameters like *r*_*m*_, *λ*, *T*, and *Dt* for bugs fed on both food sources were not statistically different, suggesting that both mature soybean pods and seeds can be suitable food sources. In the case of *H*. *halys*, all life table parameters were significantly different between the two foods sources, indicating that mature soybean pods had negative effects on their growth and reproduction. Nevertheless, those negative effects from pod feeding could be minimized from polyphagy in nature [37].

In the behavioral studies, both *R*. *pedestris* and *H*. *halys* preferred soybean seeds to pods. Hemipteran bugs can assess the quality of food substrates [38], and feeding behavior can be affected by the chemical and physical characteristics of the food substrate. Mature soybean pods have tissues more resistant to mechanical damage [39] due to the thickness of the pod wall and a higher proportion of lignin [40, 41]. Probably due to this, we observed shorter feeding times on mature soybean pods than on seeds in this study. The dense hairiness of mature soybean pods might also inhibit the movements of the bugs, and the resulting longer time preparing to feed might reduce the actual time spent feeding on mature pods. Even though both species preferred seeds to pods, few bugs (*R*. *pedestris* 0.0 and *H*. *halys* 15.6%) give up their first choice of pods, suggesting that mature soybean pods can be accepted as suitable food sources.

From both the biological and behavioral study and previous study of *H*. *halys* [42], it is clear that both species can develop and reproduce when feeding on mature soybean pods despite the hard pod shell. When we measured the thickness of the soybean pod shell, we found that the estimated depth of stylet penetration into the host tissue of both nymphs or adults of both species was much longer than the thickness of the pod shell. As these penetration estimates help identify growth stages able to penetrate the hard pod shell to reach the seed for feeding [43], this suggests that mouthpart morphology might not be a limiting factor in these bugs feeding on soybean pods.

Since it is clear that mature soybean pods with seeds inside can be an appropriate food source for both *R*. *pedestris* and *H*. *halys* despite some negative effects, bugs may cause economical damage to mature soybeans in the field. Therefore, attention should be paid to near-harvest bug management in soybean.

## Supporting information

S1 File Diet evaluation, choice response, life table parameters, and rostrum length of *Riptortus pedestris* and *Halyomorpha halys*. (XLS)
